# Inter-Individual Variability in Acute Toxicity of R-Pulegone and R-Menthofuran in Human Liver Slices and Their Influence on miRNA Expression Changes in Comparison to Acetaminophen

**DOI:** 10.3390/ijms19061805

**Published:** 2018-06-19

**Authors:** Tomáš Zárybnický, Petra Matoušková, Bibiána Lancošová, Zdeněk Šubrt, Lenka Skálová, Iva Boušová

**Affiliations:** 1 Department of Biochemical Sciences, Faculty of Pharmacy in Hradec Králové, Charles University, 500 05 Hradec Králové, Czech Republic; zarybnto@faf.cuni.cz (T.Z.); matousp7@faf.cuni.cz (P.M.); lancosob@faf.cuni.cz (B.L.); skaloval@faf.cuni.cz (L.S.); 2 Department of Surgery, Faculty of Medicine in Hradec Králové, Charles University, 500 05 Hradec Králové, Czech Republic; zdenek.subrt@fnhk.cz; 3 Department of Surgery, University Hospital Hradec Králové, 500 05 Hradec Králové, Czech Republic

**Keywords:** precision-cut liver slices, pulegone, menthofuran, acetaminophen, drug-induced liver injury, microRNA

## Abstract

Monoterpenes R-pulegone (PUL) and R-menthofuran (MF), abundant in the *Lamiaceae* family, are frequently used in herb and food products. Although their hepatotoxicity was shown in rodent species, information about their effects in human liver has been limited. The aim of our study was to test the effects of PUL, MF and acetaminophen (APAP, as a reference compound) on cell viability and microRNA (miRNA) expression in human precision-cut liver slices. Slices from five patients were used to follow up on the inter-individual variability. PUL was toxic in all liver samples (the half-maximal effective concentration was 4.0 µg/mg of tissue), while MF and surprisingly APAP only in two and three liver samples, respectively. PUL also changed miRNA expression more significantly than MF and APAP. The most pronounced effect was a marked decrease of miR-155-5p expression caused by PUL even in non-toxic concentrations in all five liver samples. Our results showed that PUL is much more toxic than MF and APAP in human liver and that miR-155-5p could be a good marker of PUL early hepatotoxicity. Marked inter-individual variabilities in all our results demonstrate the high probability of significant differences in the hepatotoxicity of tested compounds among people.

## 1. Introduction

Public interest in natural therapies, as well as global consumption of herbs and herbal products have increased significantly over past decades. Herbal medicines are considered by many as safer than evidence-based medicine, because they are regarded as “natural”. However, this approach can lead to fatal results, especially since patients do not report adverse effects of these supplements to their physicians. Therefore, the incidence of the adverse effects of herbal products is unknown and mechanisms of toxicity difficult to determine [1]. In most cases, adverse effects of natural therapies arise due to their inappropriate usage, e.g., when they are used for inappropriate indications, prepared improperly, used in large excessive dosages or for a prolonged duration of time [2]. For example, clinical cases of ingestion of large amounts of pennyroyal oil, usually used to influence dyspepsia, menorrhea and misused to induce abortion, resulted in liver damage, further described as centrilobular necrosis. Administration of pennyroyal oil to mice and rats resulted in a similar hepatotoxicity profile [1,3,4].

**Figure 1:**
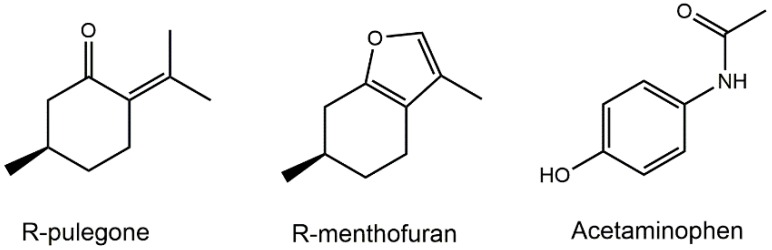
Structural formulas of studied monoterpenes and a reference compound acetaminophen.

Monoterpenes R-pulegone (PUL) and R-menthofuran (MF) (Figure 1) are major constituents of several mint (*Mentha*) species and their derived volatile oils, including peppermint (*M. piperita*), spearmint (*M. spicata*), European pennyroyal (*M. pulegium*) and American pennyroyal (*H. pulegioides*). They are used for flavoring of foods and drinks, in herbal medicinal products and cosmetics [1]. PUL was determined to be the major constituent of pennyroyal oil and MF as one of the major metabolites of PUL in the body [5]. In mice, MF showed even stronger hepatotoxicity than PUL [3]. Despite many cases of human poisoning, the toxicity of both terpenes is not well defined, and studies are usually limited to rodents. To fill this gap, the present study was designed to test the hepatotoxicity of PUL and MF in human liver using precision-cut liver tissue slices (PCLS). The main advantages of PCLS are preserved liver architecture, maintained cell communication and interaction and the possibility to use human liver pieces [6]. Moreover, the use of liver from five patients in our study allowed us to see the inter-individual variability in PUL and MF effects. In addition to the determination of the half-maximal effective concentration (EC_50_), the effect of PUL and MF on microRNA (miRNA) expression in the liver slices was also studied.

miRNAs are short (about 22 nucleotides) endogenous and non-coding RNAs, regulating gene expression post-transcriptionally by binding to mRNA, resulting in inhibition of translation or degradation of mRNA [7]. It is well known that miRNAs play a significant role in epigenetic regulation of almost all physiological and pathological processes. Nowadays, miRNAs are considered as potential biomarkers of many pathologies including liver injury. Drug-induced (or herb-induced) liver injury (DILI) is a serious and life-threatening condition, and its detection requires a suitable and optimally non-invasive biomarker [8]. Some studies indicate that certain miRNAs could be the sensitive markers of early liver injury [9,10]. For this reason, we decided to follow up on the changes in miRNA expression patterns in PCLS affected by PUL and MF in several concentrations, from non-toxic to toxic ones. Acetaminophen (APAP, see Figure 1), a well-known hepatotoxicant, was used as a reference compound to prove the functionality of our PCLS and because it is a drug with structural similarities and a liver injury phenotype close to that induced by our tested compounds. Similarly, APAP is a model drug used in studying DILI and the expression of DILI-related miRNAs. Not to mention that PCLS allow us to test toxicity and expression changes for multiple hepatotoxicants at the same time and from a single liver donor. Based on a literature review [8,11,12], eight interesting miRNAs were selected. The criteria for selection were either high expression or specificity to human liver and/or critical functions in liver physiology or pathology (Material and methods section).

Taken together, the aim of the present study was to provide new pieces of information about PUL and MF’s effects in human liver. For the first time we determined their EC_50_, PUL- and MF-induced changes in selected miRNAs expression and inter-individual differences in their effects in human liver. The presented data could contribute to the current knowledge on PUL and MF hepatotoxicity in humans and might be therefore of toxicological importance. 

## 2. Results

### 2.1. Hepatotoxicity of R-pulegone, R-menthofuran and Acetaminophen

**Figure 2:**
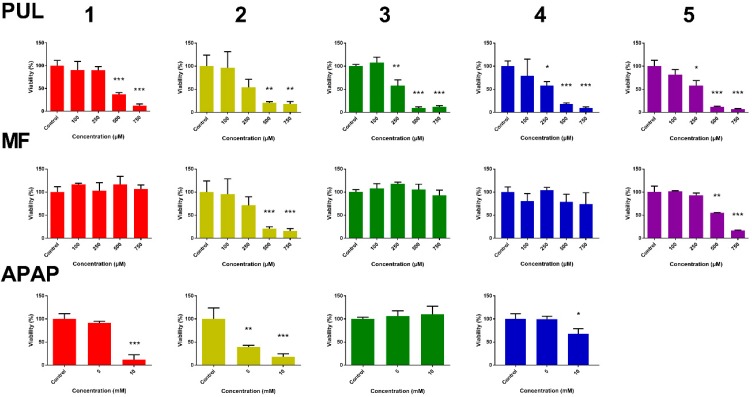
Inter-individual differences in the effect of R-pulegone, R-menthofuran and acetaminophen on viability of PCLS from five patients (1–5) after 24 h (*n* = 3), determined by ATP content. Results are presented as the mean ± SD. Statistical analyses were performed using one-way ANOVA with Dunnett’s test: *p* < 0.05 (*); *p* < 0.001 (**); *p* < 0.0001 (***).

With the aim to follow up on the hepatotoxicity of PUL, MF and APAP, their effect on viability of liver slices was measured. Adenosine triphosphate (ATP) level was used as a viability marker. In PCLS, ATP levels per mg of protein were assessed after 24 h of incubation of PLCS with studied compounds or with DMSO (untreated controls). The ATP content of the treated slices was compared to ATP content in controls, which represents 100% viability. PUL and MF toxicity was tested in PCLS from five patients, APAP as a reference compound in PCLS from four patients due to a smaller number of PCLS from the fifth patient. The obtained results are presented in Figure 2. While PUL showed a comparable toxicity in PCLS from all five patients, MF caused a significant toxicity only in PCLS from two patients. Similarly to MF, the effect of APAP showed great inter-individual variability. A significant APAP-induced decrease in PCLS viability was apparent in three of four samples treated with a higher concentration and only in one sample treated with its lower concentration.

**Figure 3:**
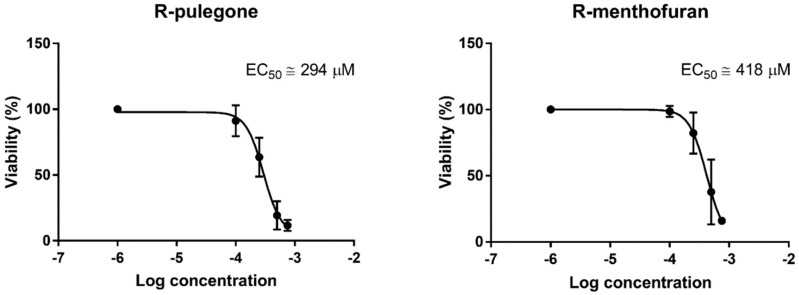
Non-linear regression of the effect of PUL and MF on the viability of PCLS and half-maximal effective concentration (EC_50_) calculation. Data represent the mean ± SD from PCLS of five liver samples (PUL) and two samples (MF) showing a significant viability decrease after the treatment.

Based on the obtained data, EC_50_ for PUL and MF were calculated via non-linear regression (Figure 3). The EC_50_ of PUL was calculated using all five liver samples, while that of MF and APAP was calculated only for those two or three samples sensitive to this compound. The EC_50_ of PUL and MF were approximately 293 µM (4.0 µg/mg of tissue) and >418 µM (>5.8 µg/mg of tissue), respectively. The APAP EC_50_ cannot be calculated from only two concentrations, but for comparison, we estimated it would be approximately >6 mM.

### 2.2. The Effect of PUL, MF and APAP on the Expression of Selected miRNAs

At the beginning of the experiment, miR-16-5p, miR-93-5p and snU6 were selected as potential reference genes. The stability of their gene expression was compared through RefFinder, a free web tool combining several other computational software, which assigns the genes a comprehensive ranking, based on their expression stability in comparison to the other tested genes (Figure S1). After further validation, miR-93-5p was chosen for our study, since its expression was the most stable under the experimental conditions. 

**Figure 4:**
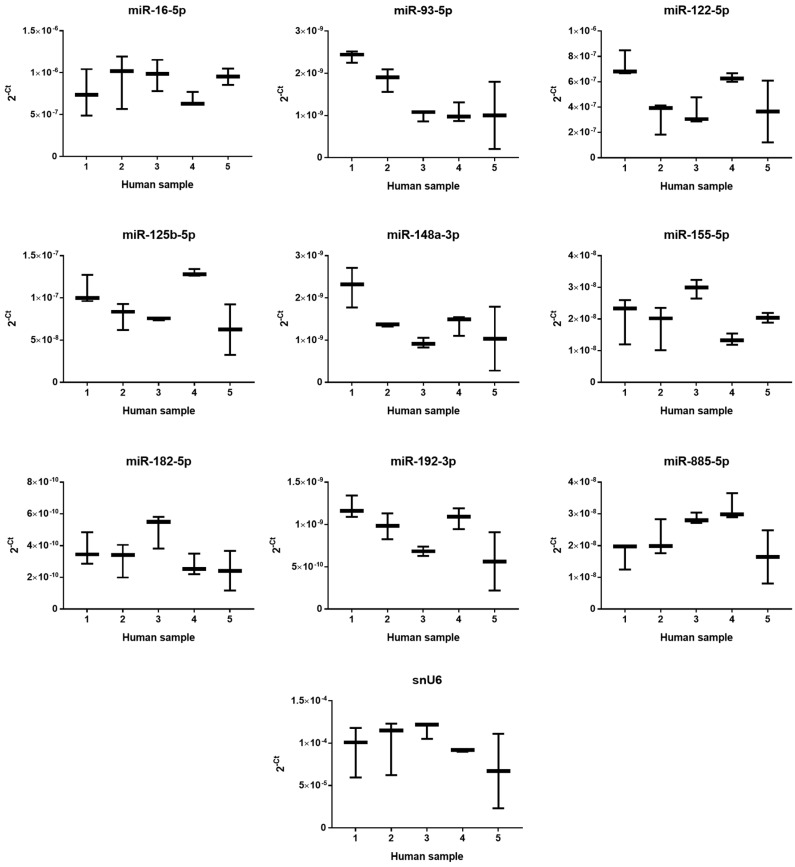
Inter-individual variability in constitutive expression of selected miRNAs in PCLS from five patients. The horizontal line represents the median, and whiskers represent the maximum and minimum values.

Based on a literature review [8,11,12], the eight most interesting miRNAs were selected. The criteria for selection were either high expression or specificity to human liver and/or critical functions in liver physiology or pathology; all criteria are summarized in Material and method section below. The constitutive expression of eight miRNAs selected for study of PUL and MF hepatotoxicity and snU6 (a frequently-used reference gene) was measured in untreated samples after 24 h of incubation using quantitative real-time polymerase chain reaction (PCR). The miRNAs’ expression was displayed as the 2^−*C*t^ for better comparison (Figure 4). The levels of individual miRNA in liver samples varied by nearly three orders of magnitude and followed the rank order: miR-182-5p < miR-93-5p ~ miR-148a-3p ~ miR-192-3p < miR-155-5p ~ miR-885-5p < miR-125b-5p < miR-122-5p < miR-16-5p. Inter-individual differences in the constitutive expression of all selected miRNAs (including relatively stable snU6) among individual liver samples were observed. Basal expression of tested miRNAs and snU6 from all five patients is summarized in Figure S2.

**Figure 5:**
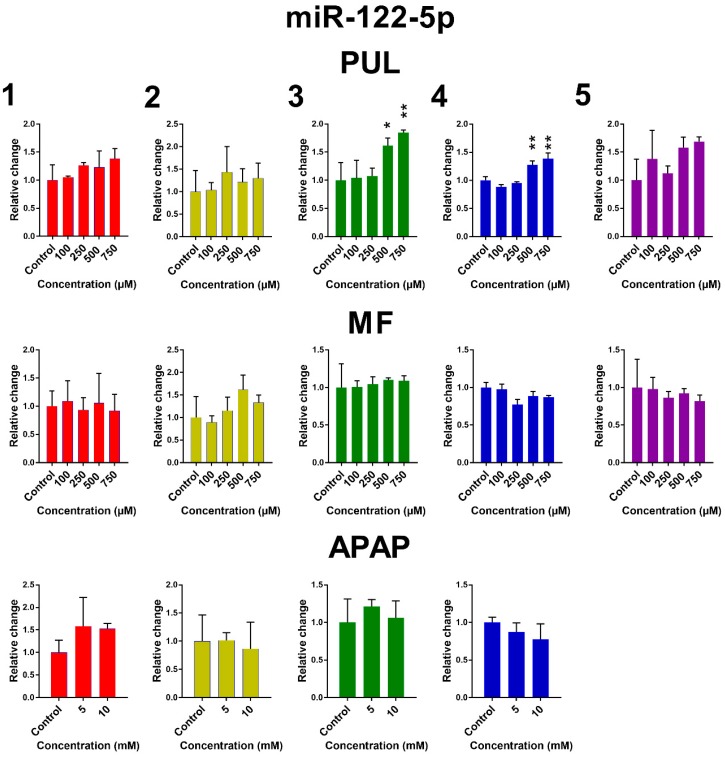

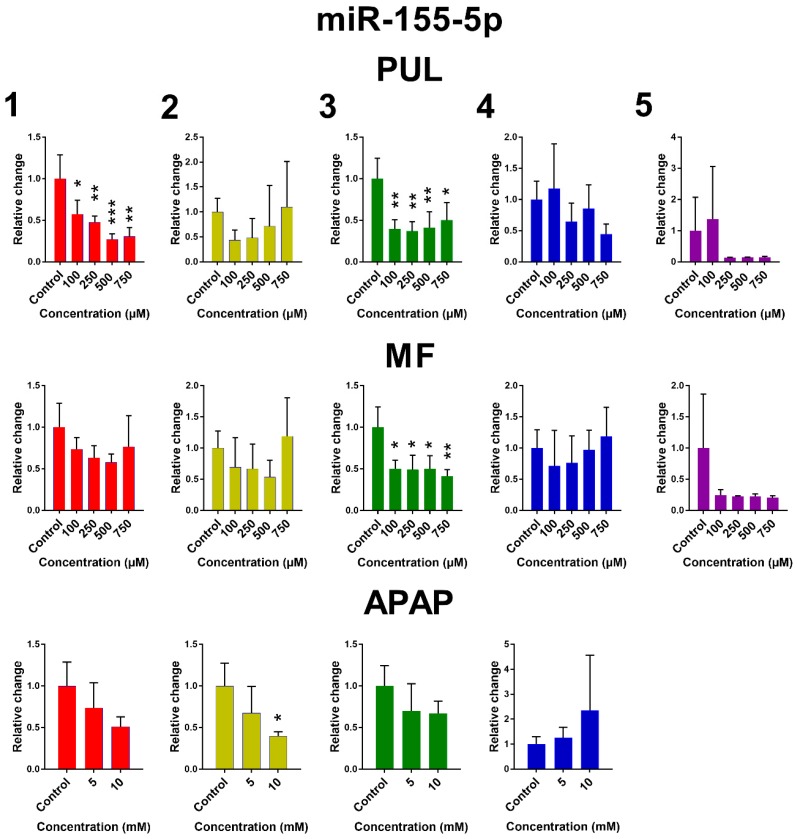

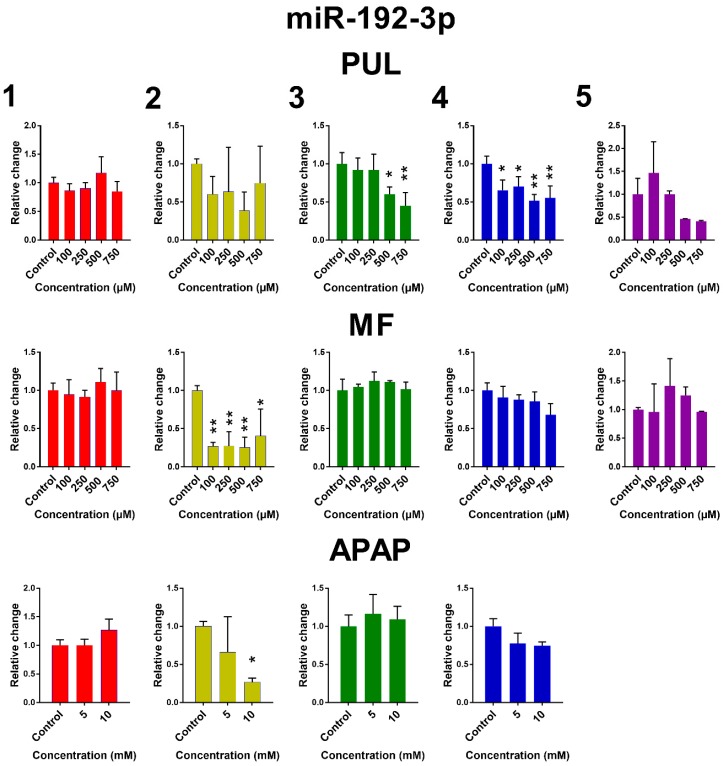
The effect of PUL, MF and APAP on the normalized expression of selected miRNAs. The normalized expression level was calculated using the 2^−ΔΔ*C*t^ method with miR-93-5p as a reference gene. Results are presented as the mean ± SD. Statistical analyses were performed using one-way ANOVA with Dunnett’s test: *p* < 0.05 (*); *p* < 0.001 (**); *p* < 0.0001 (***).

The influence of PUL, MF and APAP on the expression of miR-122-5p, miR-155-5p and miR-192-3p in PCLS is presented in Figure 5. Remaining miRNAs are presented in Figure S3. Large inter-individual differences are evident again. Although the observed effects of tested monoterpenes and APAP were rather inconsistent, some common trends in the effect of tested substances on the selected miRNA expressions can be noticed. Exposure of human PCLS to PUL significantly induced miR-122-5p expression, while levels of miR-155-5p, miR-192-3p and miR-885-5p were suppressed. PUL treatment even in non-toxic concentrations caused a marked decrease in miR-155-5p, miR-192-3p and miR-885-5p expressions in five, four and three of five liver samples, respectively, although only in two samples, this effect was statistically significant. The PUL induction effect on miR-16-5p and miR-122-5p was obvious (and significant) only at toxic concentrations. MF and APAP affected miRNAs expression to a lesser extent, and their effect was significant only in one tested liver sample.

## 3. Discussion

The monoterpenes PUL and MF are structurally-related monoterpenes. Moreover, MF is the major PUL metabolite formed via PUL 9-hydroxylation with a subsequent reduction of the carbon-carbon double bond and furan ring formation [1]. Therefore, both compounds were studied concurrently in our experiments. In addition, APAP, a drug with certain structural similarity and well-known hepatotoxicity, was included in our study, as well, as a reference compound.

In the European Union, the highest recommended daily dose of peppermint oil is 1.2 mL, and 1 g of peppermint oil contains approximately 140 mg of PUL. That is equal to 2.3 mg of PUL/kg of body weight (for a person of 60 kg) [13]. In cases of poisoning, only very few cases were examined also analytically. In the case of pennyroyal oil misuse as an abortifacient (72 h after ingestion), serum concentrations of PUL at 18 ng/mL and MF at 1 ng/mL detectable 26 h post-mortem resulted in fatal poisoning. In another case, 10 h after ingestion, only MF in the concentration of 40 ng/mL was detected [14]. Therefore, it is very difficult to estimate the possible concentration of PUL and MF in the human body (and not at all in liver). Similarly, it is also difficult to estimate the hepatotoxicity of PUL and MF to humans as their effects have been studied mostly in rodent species. The intra-peritoneal application of pennyroyal oil to mice and rats resulted in a hepatic toxicity profile comparable to that in humans [3,15,16]. In mice, a dose greater than 100 mg/kg resulted in increased levels of plasmatic aminotransferases and decreased levels of liver glutathione [1,3]. Although PUL was identified as the major toxic compound present in pennyroyal oil, MF exerted higher toxicity in mice [3]. The stereochemistry plays a considerable role, as well, since S-enantiomer of pulegone was described to be about three-times less toxic than R-enantiomer [3]. In rats, oral administration of PUL (400 mg/kg) and MF (250 mg/kg) resulted in a significant decrease in the levels of liver microsomal cytochrome P450 and massive hepatotoxicity [4,17]. Despite a large effort, the results from animal studies are difficult to translate to humans, since the route of administration, time of exposure and different animal species or strain can all influence the obtained results significantly.

With respect to well-known inter-species differences in xenobiotics’ toxicity, we decided to test PUL and MF hepatotoxicity in PCLS and compare it with APAP. In addition to cell viability, expression of selected miRNAs was also studied, since miRNAs are now considered as potential markers of liver injury [18]. The PCLS from five patients were used to follow up on the possible inter-individual differences in sensitivity toward toxicants.

Our results clearly demonstrate PUL hepatotoxicity in PCLS from all five patients. In the case of MF, only two out of five liver samples were sensitive towards its exposure in tested doses. Furthermore, APAP, a well-known hepatotoxicant, was not toxic in one liver sample. There are many possible explanations for such large inter-individual differences in sensitivity toward the tested compounds, e.g., different activity of drug-metabolizing enzymes, antioxidant enzymes and endogenous antioxidants, which all could be affected by pathologies, medication, food, environmental exposition, etc. The influence of cytochrome P450 inducers/inhibitors on PUL and MF toxicity or metabolites’ ratio production was proven in both in vitro [19,20] and in vivo [3,5] studies.

The calculation of EC_50_ showed that PUL is more toxic than MF in human liver. This result was surprising because MF (as a major metabolite of PUL) has been considered responsible for PUL toxicity based on the previous animal in vivo studies [1,5]. An in vivo study investigating the MF contribution to PUL hepatotoxicity stated that MF contributed to approximately 50% of total PUL toxicity in rats [15]. The only in vitro study focused on MF toxicity was performed using rat PCLS and showed time- and concentration-dependent loss of intracellular lactate dehydrogenase after 6 h of incubation with MF (0.1–1 mM) [20]. Despite that, their PCLS handling certainly did not allow keeping the slices viable even up to 24 h; therefore, the sensitivity towards MF shall be questioned. Our results indicate that human liver sensitivity towards MF is lower than the sensitivity of rat liver. However, also another possibility exists, that MF with its reactive furan ring is rapidly metabolized on the periphery of PCLS, before influencing all of the cells in PCLS. Higher doses of peppermint oil, in which PUL is one of the active constituents, caused a decrease in human hepatoma HG2P128 cells’ viability in vitro [21]. In human hepatocytes and HepaRG cells, PUL was shown to produce glutathione-trapped reactive metabolites, which may be responsible for its idiosyncratic toxicity [22].

In our study, APAP was used as a reference compound. APAP is often used as a model drug for drug-induced liver injury studies. Despite that, the estimation of the EC_50_ of APAP in human liver is complicated due to interspecies differences [23,24] and also individual differences in sensitivity caused by divergent gene expression, activity of drug-metabolizing enzymes or liver glutathione content [25,26], as well as technical issues (e.g., heterogeneous handling, culture setup or incubation system, protocols) [27]. Based on the literature review, concentrations of APAP in our study used were 5 mM (expecting none or slight toxicity) and 10 mM (expecting moderate to severe toxicity) [26,27,28]. The obtained results showed APAP toxicity in human PCLS, although one liver sample was insensitive to APAP at all tested concentrations. Moreover, approximate EC_50_ for APAP was much higher than that of PUL, which demonstrates high PUL toxicity in human liver.

The second objective of our study was to follow up on the effects of PUL, MF and APAP on the expression of selected miRNAs using real-time quantitative PCR. This method is sensitive and reproducible; however, the results depend on proper normalization entirely [29]. For this reason, the adequate reference gene was searched for. snU6, a small nuclear RNA, is a frequently-used reference gene for miRNAs expression semi-quantification. Although snU6 is also a non-coding RNA, it is not an miRNA, and its synthesis pathway is different and does not reflect the biochemical character of miRNA, so high caution needs to be taken [30,31]. Indeed, the comparison of snU6 expression in five liver samples revealed great inter-individual differences. From all three tested reference genes, miR-93-5p was found to be the most stable one; hence, it was used for further data normalization.

Our miRNA expression results showed high variability, although it can be partially expected, from the large variability in the toxicity results, especially for MF and APAP. Moreover, the comparison of constitutive expression of selected miRNAs also revealed big differences among individual liver samples, which may reflect different physiological/pathological conditions. In the literature, we can find significant alterations of gene expression levels in the tissue between healthy and sick/injured populations; however, this is just a relative comparison, and the physiological range of miRNA expression levels has not been determined yet. Although some inter-individual differences in basal levels of selected miRNAs can be seen in the presented experiments, it is possible that these levels are still within the physiological range. Nevertheless, PUL significantly changed the expression of certain miRNAs in most liver samples. Interestingly, the PUL-mediated decrease of miR-155-5p and miR-885-5p was more pronounced in liver samples with higher constitutive expression of these miRNAs and vice versa; in Sample 3 with lower constitutive expression of miR-122-5p, the increase of this miRNA after PUL treatment was the most significant. Downregulation of miR-122 was found in the liver of patients suffering various types of liver cancer including cholangiocellular carcinoma. It has been reported that miR-122 overexpression can suppress cholangiocarcinoma cell invasion and migration [32]. However, miR-155-5p, which exhibited decreased expression by PUL even in a non-toxic concentration, was affected in all liver samples. This miRNA appears to be one of the most biologically-relevant miRNAs in several liver diseases and seems to exert pleiotropic functions depending on the etiology and disease context. Ambiguous roles are found in non-viral diseases: miR-155 knockout mice are protected from Fas-induced liver injury by activating the anti-apoptotic survival factor MCL-1 (myeloid cell leukemia 1) [33]. Moreover, miR-155-5p is probably involved in the regulation of lipid metabolism, as its hepatic expression was increased in mice with non-alcoholic fatty liver disease [34,35]. In mice, miR-155, a regulator of inflammation, promoted alcohol-induced steatohepatitis and liver fibrosis, while miR-155 knockouts were protected [36]. The potential use of miR-155-5p as a biomarker of PUL toxicity deserves further attention.

Despite the patients being under different clinical conditions, liver samples for experiments were selected to be as healthy as possible. Plasma levels of aspartate aminotransferase (0.35–0.46 µkat/L), alanine aminotransferase (0.31–0.51 µkat/L), bilirubin (5–9.7 µkat/L) and alkaline phosphatase (0.61–1.2 µkat/L), which provide information about liver (dys)functions, occurred within the physiological range in all five patients. The level of γ-glutamyltransferase (1.13–3.5 µkat/L) was the only elevated parameter in all five patients. Elevation in γ-glutamyltransferase levels is often seen in patients with colorectal carcinoma with liver metastasis [37] or in patients with biliary tract diseases including malignancies [38]. All liver samples were also scored by the pathologist for steatosis (score of 0–1) and fibrosis (score of zero), and the obtained scores indicate no or mild signs of liver disease in patients’ biopsies.

Pharmacotherapy could influence the obtained results, as the effects of drugs administered alone or in combination on miRNA expression are not fully understood yet. On the other hand, liver functions in patients with different types of malignancy affecting directly or indirectly liver need to be well monitored, and miRNA expression level assessment may be applied in these individuals in the future. For this reason, human tissues should be used in experiments in order to discover, describe and define inter-/intra-individual factors that will allow normalization and application of miRNA markers into clinical practice.

In conclusion, our data allowed us to calculate and compare for the first time the half-maximal effective concentration of monoterpenes PUL and MF for PCLS. Moreover, the influence of tested monoterpenes on the expression of certain miRNAs was shown. Our results obtained in human liver differed from previous results from animal studies, which emphasize the necessity of toxicological tests in human models. In all experiments, large inter-individual differences were observed, which should be taken into account in all toxicity evaluation studies.

## 4. Materials and Methods 

### 4.1. Chemicals and Reagents

All chemicals were obtained from Sigma Aldrich (Prague, Czech Republic) unless stated otherwise. Stock solutions were prepared in dimethyl sulfoxide (DMSO) and stored at 4 °C in the dark. The final DMSO concentration did not exceed 0.2%.

### 4.2. Ethics Statement

The Ethics Committee of University Hospital in Hradec Králové, Czech Republic (Permission No. 201703 S14P, 2 March 2017) approved all the procedures. All patients signed an informed consent for tissue procurement for research purposes.

### 4.3. Human Liver

Small pieces of healthy liver tissue were obtained from five patients (1 male, 4 females, 57–73 years old) undergoing partial hepatectomy due to a tumor. Liver samples were instantly put into a chilled vessel with Euro-Collins solution and transported to the laboratory for the immediate preparation of precision-cut liver slices. The characteristics of study subjects who provided liver tissue are listed in Table 1. Liver biopsies were considered as healthy based on the results of routine biochemical tests performed prior to surgery (plasma levels of bilirubin, alanine aminotransferase, aspartate aminotransferase, γ-glutamyltransferase and alkaline phosphatase) and on the histological judgement of the pathologist on liver steatosis and fibrosis. Plasma levels of all assessed enzymes were within physiological range except for γ-glutamyltransferase, which was 1.7–5.2× elevated above the physiological range in all five patients. Histological scoring for steatosis and fibrosis was performed by the pathologist, and all liver biopsies received a score of 0–1 for steatosis and 0 for fibrosis, indicating none or mild signs of liver disease.

### 4.4. Precision-Cut Liver Slice Preparation and Experimental Treatment

Liver tissue was preserved in Euro-Collins solution until use. Cores with a diameter of 8 mm were punched out of the tissue. Liver slices were prepared in ice-cold Krebs–Henseleit buffer supplemented with 25 mM d-glucose, 25 mM NaHCO_3_, 10 mM HEPES (Carl Roth, Karlsruhe, Germany) and saturated with carbogen (95% O_2_, 5% CO_2_) using a Krumdieck tissue slicer MD4000 (Alabama Research and Development, Munford, AL, USA). PCLS (diameter: 8 mm, thickness: 150–170 μm) were incubated individually in Williams’ Medium E (with l-glutamine, Invitrogen, Paisley, UK) supplemented with 25 mM glucose (final concentration 36 mM) and 50 μg/mL gentamycin at 37 °C under continuous supply of 85% O_2_/5% CO_2_ in 12-well plates with continuous shaking (90 times/min). After 1 h of preincubation in 1 mL of medium, the slices were transferred to new 12-well plates with 1.3 mL of fresh medium and subsequently incubated for 24 h in the presence or absence of tested compounds. All experiments were performed in triplicate using liver tissue from five different patients. The wet weight of slices was approximately 14.4 ± 2.1 mg.

### 4.5. ATP Determination

The viability of the slices was determined by measuring ATP levels [39]. The slices were collected separately; each slice was put into 1 mL of ethanol solution (70% (*v*/*v*) containing 2 mM ethylenediaminetetraacetic acid, pH 10.9), immediately frozen and stored at −80 °C until further analyses. After thawing, the slices were homogenized with the FastPrep-24 5G Instrument (MP Biomedicals, Santa Ana, CA, USA) and centrifuged for 5 min at 12,000× *g* at 4 °C. ATP content was measured in supernatant using the ATP Bioluminescence Assay Kit CLS II (Roche, Mannheim, Germany) in a black 96-well plate according to the manufacturer's protocol using plate reader Tecan Infinite M200 (Tecan Group, Männedorf, Switzerland) and a standard ATP calibration curve. The concentration of ATP was corrected for the total protein content from the remaining sample pellet. The sample pellet was dissolved in 200 μL of 5 M NaOH for 30 min at 37 °C after dilution by water to 1 M NaOH. The protein content was estimated using the BCA assay kit, using bovine serum albumin for the calibration curve.

### 4.6. Gene Expression

Total RNA was isolated using TriReagent according to the manufacturer’s instructions (Molecular Research Center, Cincinnati, OH, USA). RNA yields and purity were determined measuring the absorbance at 260 and 280 nm using NanoDrop ND-1000 UV-Vis Spectrophotometer (Thermo Fisher Scientific, Pardubice, Czech Republic). In order to avoid genomic DNA contamination, 4 µg of RNA were treated with DNase I (New England Biolabs, Ipswich, MA, USA) for 20 min at 37 °C, inactivated by heat (10 min at 75 °C) and diluted to a concentration of 0.1 µg/µL. RNA was stored at −80 °C until further analyses. First strand cDNA was synthesized from 250 ng of total RNA using ProtoScript II reverse transcriptase (New England Biolabs, Ipswich, MA, USA). The reaction mixture (10 µL) included stem-loop oligonucleotides specific for each miRNA and U6. After initial heat denaturation of total RNA (65 °C for 5 min), the reactions were incubated for 30 min at 16 °C, for 30 min at 42 °C and for 5 min at 95 °C. Obtained cDNAs were diluted 12.5×. The qPCR analyses were performed using QuantStudio 6 Flex (Applied Biosystems, Foster City, CA, USA) using the Xceed qPCR SG Mix (Institute of Applied Biotechnologies, Prague, Czech Republic) following the manufacturer’s protocol. Primer sequences are listed in Table 2. Relative expression levels were calculated as fold change using the 2^−ΔΔ*C*t^ method [40].

### 4.7. Statistics

In each experiment, slices in triplicate were used for each condition, and each slice was analyzed separately. Results are expressed as the mean ± SD. Comparisons among multiple groups were performed using one-way analysis of variance (ANOVA) with Dunnett’s test, using GraphPad Prism 7 (GraphPad Software, La Jolla, CA, USA). Differences were considered as significant when *p* < 0.05.

## Figures and Tables

**Table 1:** Summary of human samples.

Human Sample	Gender (Age)	Reason of Surgery	Long-Term Pharmacotherapy
1	Male (69)	Colorectal carcinoma	Acetylsalicylic acid, nitrendipine
2	Female (73)	Colorectal carcinoma	Lercanidipine, furosemide, perindopril, nadroparin
3	Female (67)	Cholangiocellular carcinoma	Nadroparin
4	Female (65)	Colorectal carcinoma	Amlodipine
5	Female (57)	Colorectal carcinoma with liver metastasis	none

**Table 2:** List of selected genes, their functions and sequences of primers used.

Gene	Primer Sequences 5′-3′	Function
hsa-miR-16-5p	F: ACAGCCTAGCAGCACGTAAAT RT: GTCTCCTCTGGTGCAGGGTCC GAGGTATTCGCACCAGAGGAGACC GCCAA	Modulation of expression alters hepatic stellate cells’ autophagy, reference gene [41,42,43]
hsa-miR-93-5p	F: GTCAATCAAAGTGCTGTTCGTG RT: GTCTCCTCTGGTGCAGG GTCCGAGGTATTCGCACCAGAGGAGAC	Aberrant expression probably plays a role in hepatoma development, reference gene [42,44,45,46]
hsa-miR-122-5p	F: AGACCTCCTGTGCAAATCTATG RT: GTCTCCTCTGGTGCAGGGTCC GAGGTATTCGCACCAGAGGAGACC AAACA	Control of diverse aspects of hepatic function (lipid metabolism) or dysfunction (viral infection, hepatocarcinogenesis), potential liver injury biomarker [47,48,49,50]
hsa-miR-125b-5p	F: AGACCTCCTGTGCAAATCTATG RT: GTCTCCTCTGGTGCAGGGTCC GAGGTATTCGCACCAGAGGAGACC AAACA	Tumor suppressor, potential diagnostic tool for hepatitis B virus-induced hepatocellular carcinoma [51,52]
hsa-miR-148a-3p	F: GAGAATTCAGTGCACTACAGA RT: GTCTCCTCTGGTGCAGGGTCC GAGGTATTCGCACCAGAGGAGACA CAAAG	Downregulated in hepatocellular carcinoma, deficiency enhances hepatic steatosis [53]
hsa-miR-155-5p	F: GGCCCTTTAATGCTAATCGTGA RT: GTCTCCTCTGGTGCAGGGTCC GAGGTATTCGCACCAGAGGAGACA CCCCT	Important role in immune and inflammatory processes, oncogenic [54,55]
hsa-miR-182-5p	F: GATCACTTTGGCAATGGTAGAAC RT: GTCTCCTCTGGTGCAGG GTCCGAGGTATTCGCACCAGAG GAGACAGTGTG	Attenuates liver ischemia-reperfusion injury, plays a role in hepatocellular carcinoma and its metastasis [56,57,58]
hsa-miR-192-3p	F: ACGTGTCTGCCAATTCCATAG RT: GTCTCCTCTGGTGCAGGGTCC GAGGTATTCGCACCAGAGGAGACT ATTTA	Suppresses farnesoid X receptor expression in adenocarcinoma cell lines [59]
hsa-miR-885-5p	F: GAGACATCCATTAC CTACCC RT: GTCTCCTCTGGTGCAGGGTCC GAGGTATTCGCACCAGAGGAGACA GAGGC	Lipoprotein and lipid metabolism, suppression of hepatocellular carcinoma metastasis, potential liver injury biomarker [60,61,62,63]
snU6	F: GCTCGCTTCGGCAGCACA RT: AACGCTTCACGAATTTGCGTG	Reference gene [64]
Universal	R: GAGGTATTCGCACCAGAGGA	

## Supplementary Materials

Supplementary materials can be found at http://www.mdpi.com/1422-0067/19/6/1805/s1.

## Abbreviations

APAP	acetaminophen
ATP	adenosine triphosphate
DILI	drug-induced liver injury
EC_50_	half-maximal effective concentration
MF	R-menthofuran
miRNA	microRNA
PCLS	precision-cut liver slices
PUL	R-pulegone
qPCR	quantitative polymerase chain reaction
