# Supplementary material for: Inter-Individual Variability in Acute Toxicity of R-Pulegone and R-Menthofuran in Human Liver Slices and Their Influence on miRNA Expression Changes in Comparison to Acetaminophen
Source: Int J Mol Sci. 2018 Jun 19;19(6):1805. doi: 10.3390/ijms19061805 (PMC6032148; doi:10.3390/ijms19061805)
Supplement: Supplementary file 1 [file ijms-19-01805-s001.pdf]

Inter-Individual Variability in Acute Toxicity of R-Pulegone and R-Menthofuran in Human Liver Slices and Their Influence on miRNA Expression Changes in Comparison to Acetaminophen

S1. Results

S1.1. The Effect of PUL, MF and APAP on the Expression of Selected miRNAs

At the beginning of the experiment, miR-16-5p, miR-93-5p and snU6 were selected as potential reference genes. The stability of their gene expression was compared through RefFinder, a free web tool combining several other computational software, which assigns the genes a comprehensive ranking, based on their expression stability in comparison to the other tested genes (Figure S1). After further validation, miR-93-5p was chosen for our study, since its expression was the most stable under the experimental conditions.

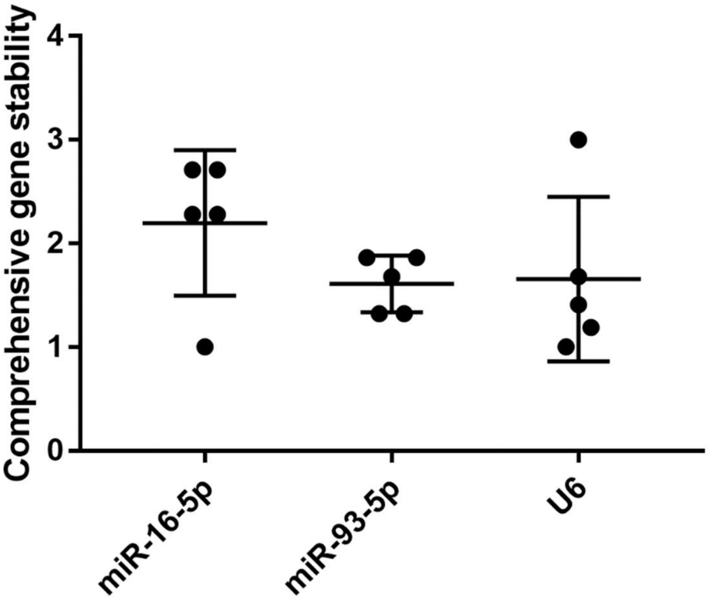

Figure S1. RefFinder analysis of candidate reference genes for miRNA normalization.

The constitutive expression of eight miRNAs selected for study of PUL and MF hepatotoxicity and snU6 (frequently used reference gene) was measured in untreated samples after 24 hours incubation using quantitative real-time PCR. The miRNAs and snU6 expression was displayed as the 2^{-Ct} for better comparison. The levels of individual miRNA in liver samples varied by nearly three orders of magnitude and followed the rank order: miR-182-5p < miR-93-5p ~ miR-148a-3p ~ 192-3p < miR-155-5p ~ miR-885-5p < miR-125b-5p < miR-122-5p < miR-16-5p. Levels of basal expression of tested miRNAs and snU6 from all five patients are summarized in Figure S2. The highest and the lowest constitutive expression had snU6 and miR-182-5p, respectively.

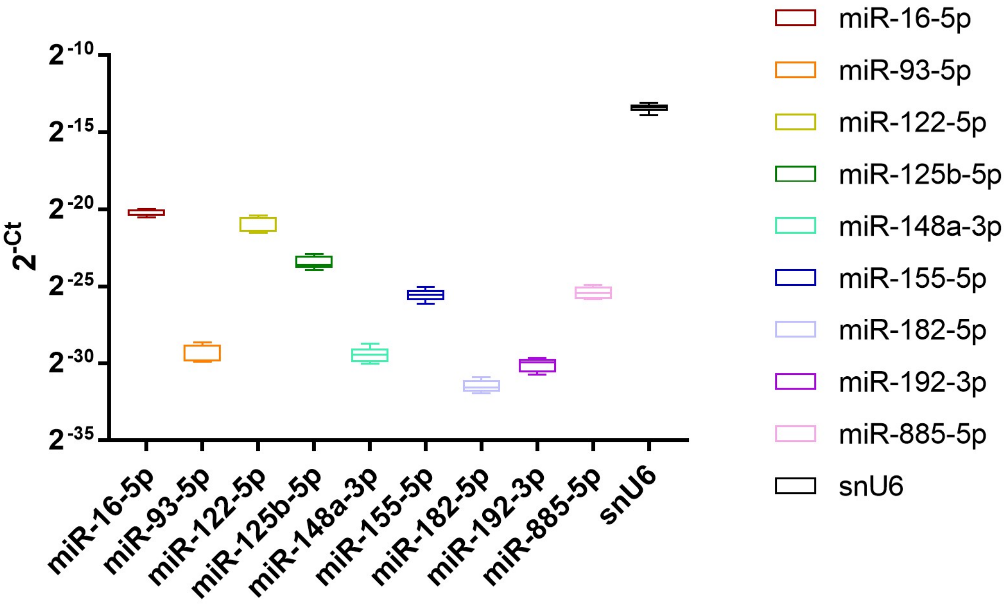

Figure S2. Box-and-whisker plot of median constitutive miRNA and snU6 expression in PCLS from five patients. The bottom and top of the box represent the 1st and 3rd quartiles, the line within the box represents the median value and whiskers represent the maximum and minimum values.

The influence of PUL, MF and APAP on the expression of miR-122-5p, miR-155-5p and miR-192-3p in PCLS is presented in Figure 5. Remaining miRNAs are presented in Figure S3. Large inter-individual differences are evident again. Although the observed effects of tested monoterpenes and APAP were rather inconsistent, some common trends in the effect of tested substances on the selected miRNA expressions can be noticed. Exposure of human PCLS to PUL significantly induced miR-122-5p expression, while levels of miR-155-5p, miR-192-3p and miR-885-5p were suppressed. PUL treatment even in non-toxic concentrations caused marked decrease in miR-155-5p, miR-192-3p and miR-885-5p expressions in five, four and three of five liver samples, respectively, although only in two samples this effect was statistically significant. PUL induction effect on miR-16-5p and miR-122-5p was obvious (and significant) only in toxic concentrations. MF and APAP affected miRNAs expression to lesser extend and their effect was usually significant only in one tested liver sample.

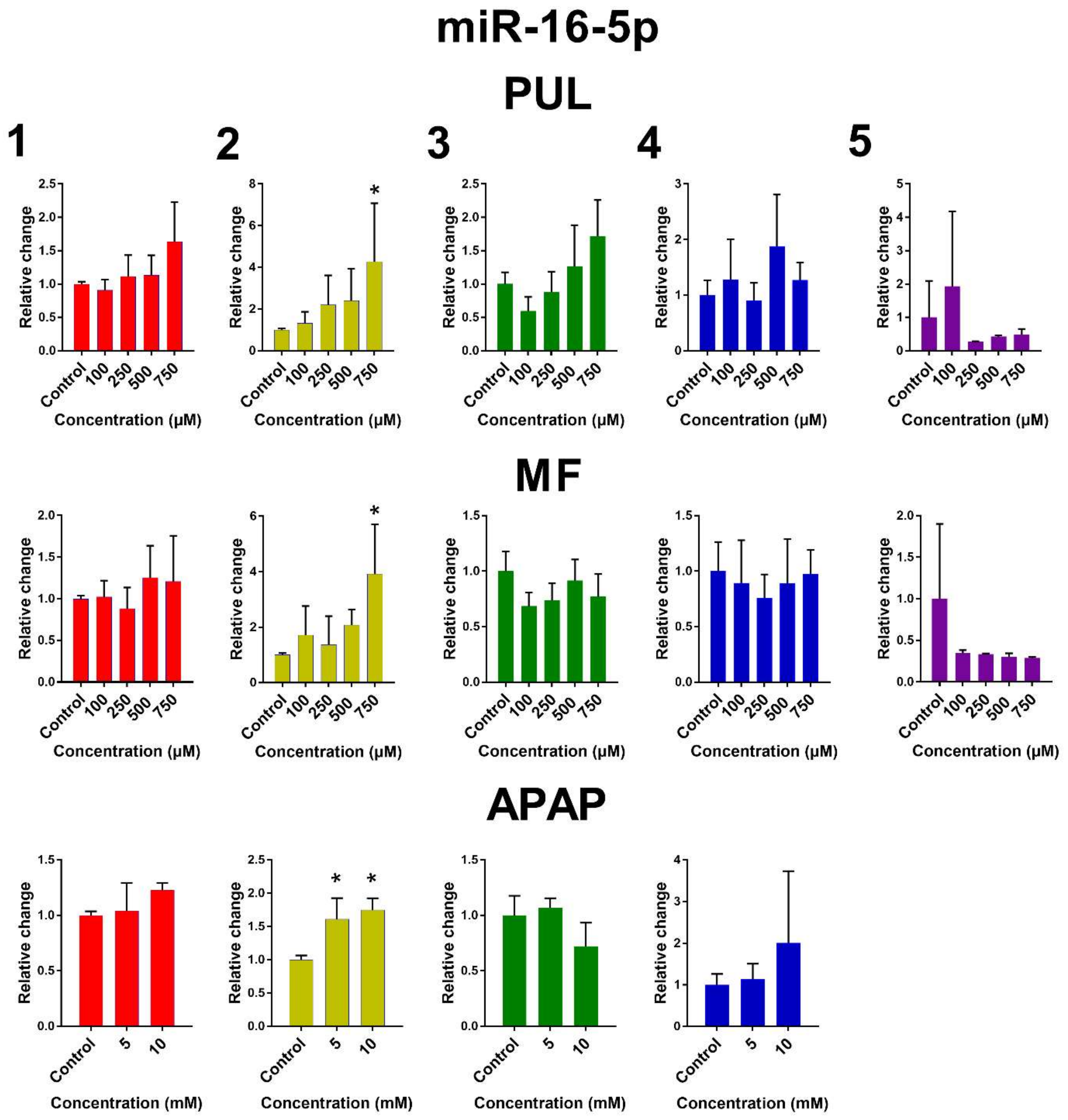

miR-16-5p
PUL
1
2
3
4
5
MF
APAP
Relative change
Concentration (µM)
Concentration (mM)
Control
100
250
500
750
5
10
*

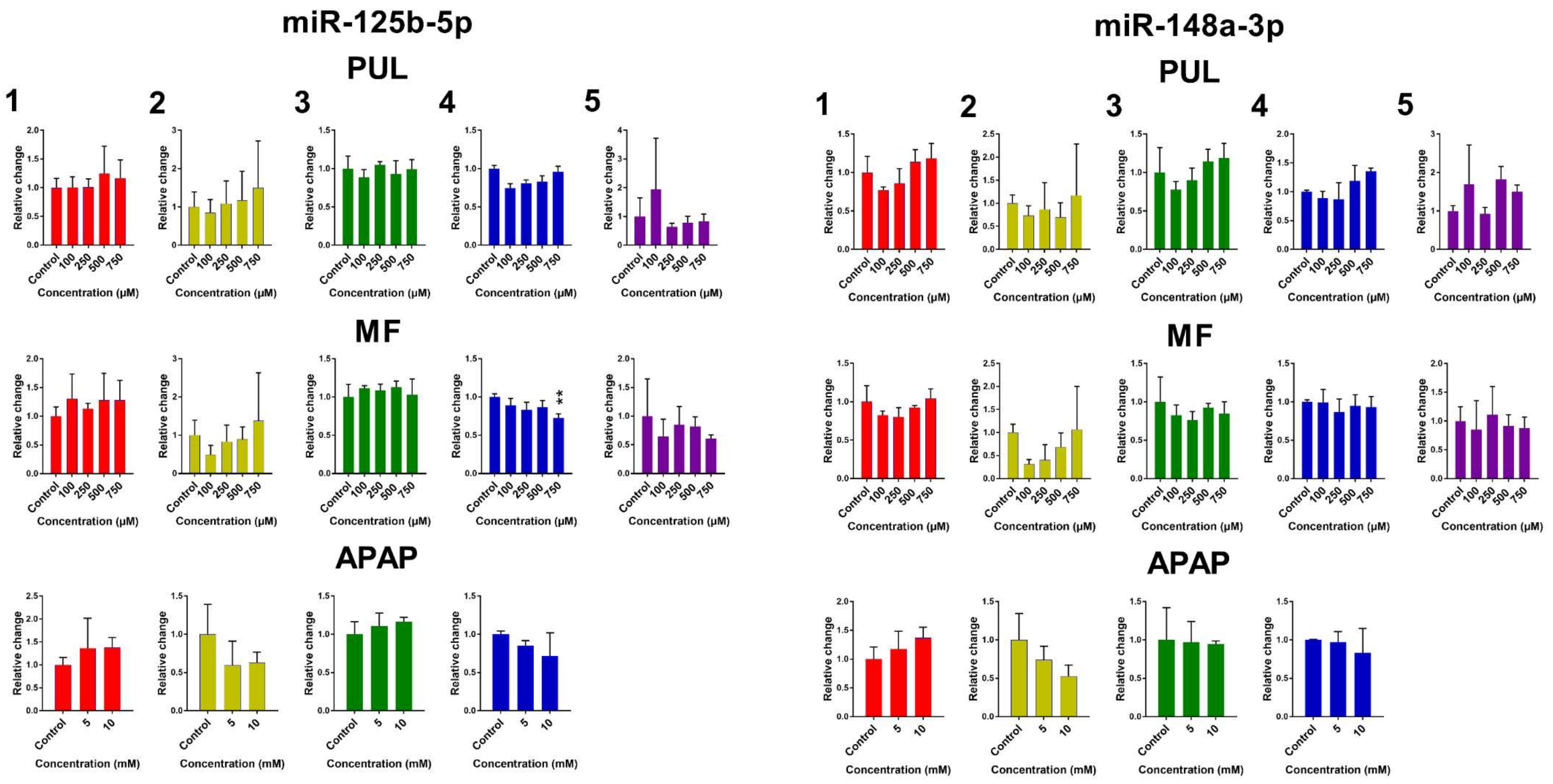

miR-125b-5p
PUL
MF
APAP
miR-148a-3p
PUL
MF
APAP
1
2
3
4
5
Relative change
Concentration (µM)
Concentration (mM)
Control
100
250
500
750
5
10
**

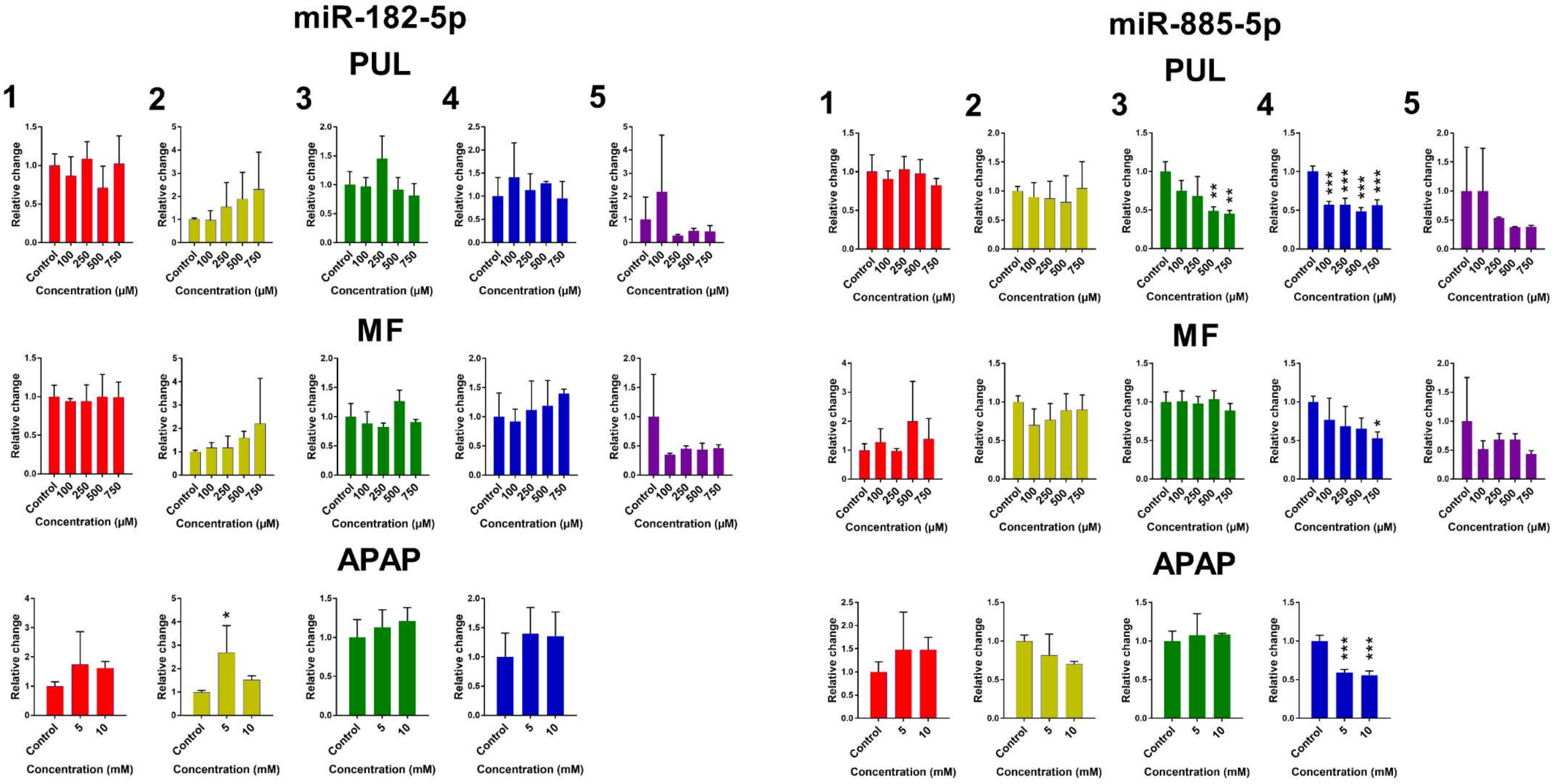

Figure S3. The effect of R-pulegone, R-menthofuran and acetaminophen on the normalized expression of selected miRNAs. The normalized expression level was calculated using the $2^{-\Delta\Delta Ct}$ method with miR-93-5p as a reference gene. Results are presented as the mean ± SD. Statistical analyses were performed using one-way ANOVA with Dunnett's test: $p < 0.05$ (*); $p < 0.001$ (**); $p < 0.0001$ (***).
